# Role of respiratory system microbiota in development of lung cancer and clinical application

**DOI:** 10.1002/imt2.232

**Published:** 2024-08-25

**Authors:** Bowen Li, Daoyun Wang, Chengye Zhang, Yadong Wang, Zhicheng Huang, Libing Yang, Huaxia Yang, Naixin Liang, Shanqing Li, Zhihua Liu

**Affiliations:** ^1^ Department of Thoracic Surgery Peking Union Medical College Hospital, Chinese Academy of Medical Sciences and Peking Union Medical College Beijing China; ^2^ Institute for Immunology, School of Basic Medical Sciences Tsinghua University Beijing China; ^3^ Department of Rheumatology and Clinical Immunology Peking Union Medical College Hospital, Chinese Academy of Medical Sciences and Peking Union Medical College Beijing China; ^4^ Tsinghua‐Peking Center for Life Sciences Beijing China

## Abstract

Microbes play a significant role in human tumor development and profoundly impact treatment efficacy, particularly in immunotherapy. The respiratory tract extensively interacts with the external environment and possesses a mucosal immune system. This prompts consideration of the relationship between respiratory microbiota and lung cancer. Advancements in culture‐independent techniques have revealed unique communities within the lower respiratory tract. Here, we provide an overview of the respiratory microbiota composition, dysbiosis characteristics in lung cancer patients, and microbiota profiles within lung cancer. We delve into how the lung microbiota contributes to lung cancer onset and progression through direct functions, sustained immune activation, and immunosuppressive mechanisms. Furthermore, we emphasize the clinical utility of respiratory microbiota in prognosis and treatment optimization for lung cancer.

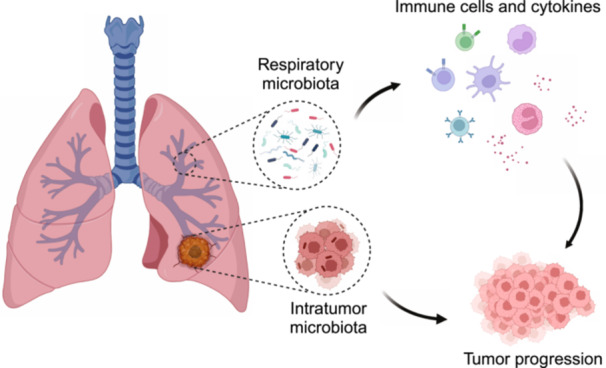

Microbiomes have been demonstrated to play a profound role in tumor development, thus being proposed as one of the emerging “Hallmarks of Cancer.” Microorganisms of the greatest attention are gut bacteria, with specific species found to promote tumor development and impact the efficacy of treatments, particularly immunotherapy. This effect may stem from the close interaction between bacteria and the immune system, both locally and systemically. Akin to the intestine, the respiratory tract extensively interacts with the external environment and possesses a mucosal immune system. This prompts consideration of the relationship between respiratory microbiota and lung cancer.

## RESPIRATORY MICROBIOTA

### Composition

**Figure 1:**
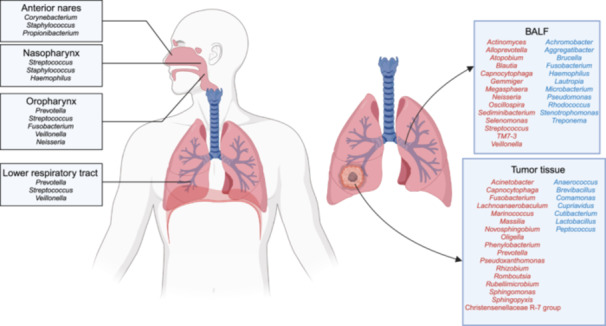
Resident microbiota of the normal human respiratory tract (left) and the impact of lung cancer on the lung microbiota (right). The text box titled “BALF” (abbreviation for bronchoalveolar lavage fluid) indicates the changes in the lower respiratory tract microbiota in lung cancer patients compared with healthy individuals or those with benign lung diseases, measured by BALF. The text box titled “Tumor tissue” indicates the changes in the microbiota of lung cancer tissues compared with adjacent normal lung tissues. Red denotes an increase in abundance, while blue denotes a decrease in abundance.

Niche‐specific bacterial communities inhabit the surface of the adult respiratory tract with a roughly decreasing trend in bacterial burden from the upper to lower respiratory tract. Two bacterial phyla, *Actinobacteria* and *Firmicutes*, are the predominant settlers in the anterior nares [1]. In the nasopharynx, there is a notable elevation in the relative abundance of the phyla *Firmicutes* and *Proteobacteria*, resulting in their predominance as colonizers, while the proportion of *Actinobacteria* diminishes [2]. In the oropharynx, *Bacteroidetes* emerge as the predominant phylum (Figure 1) [2].

The bacterial load in the lower respiratory tract of healthy individuals is significantly lower than that in the upper respiratory tract, to the extent that it was previously considered sterile. Compared with the upper airway, the bacterial burden in the lower airway is lower by 100–10,000 times. The microbial communities in the lower respiratory tract and lung are predominantly composed of bacteria belonging to the phyla *Firmicutes*, *Bacteroidetes*, and *Proteobacteria*. Specifically, the prevalent genera include *Prevotella*, *Streptococcus*, and *Veillonella* (Figure 1) [3]. It is also crucial to acknowledge that respiratory bacteria are significantly influenced by environmental factors and individual physiological states, such as seasons, climate, pollution, age, smoking, gender, antibiotic usage, and even pet ownership.

16 S rRNA sequencing, the most commonly used technique for studying lung microbiota, is based on amplifying and then sequencing the variable regions of the bacterial 16 S rRNA genes. Metagenomics, another genomic analysis of microbes, employs a genome‐wide shotgun sequencing approach. It should be noted that much of the bacterial DNA detected in the lungs may originate from nonviable bacteria, as a study using porcine lungs has revealed that over 90% of microbial DNA is sensitive to DNase I [4]. Thus, it is important to keep in mind that bacterial abundance and diversity could be overestimated in the lower respiratory tract.

### Microbial dysbiosis in lung with tumor

The lung microbiota of patients with lung cancer exhibits alterations compared with that of healthy individuals, a phenomenon known as microbial dysbiosis. Due to substantial individual variation and limited sample sizes, studies on changes in the lower respiratory tract microbiota composition in lung cancer patients have reached conclusions that are not entirely consistent. Among these findings, it is relatively certain that the relative abundance of the phyla *Firmicutes* and *TM7* increases, while *Proteobacteria* decreases [5]. At the genus level, although *Prevotella* remains the dominant lung microbiota in lung cancer patients, its abundance significantly decreases, while *Veillonella* becomes more abundant (Figure 1) [5].

The pathological type, stage, and other clinical characteristics of lung cancer can influence the composition of the lung microbiota. Notably, a study categorized patients’ lung microbiota composition into two pneumotypes: one enriched with background predominant taxa (BPT), such as *Flavobacterium* and *Pseudomonas*, and another enriched with supraglottic predominant taxa (SPT), such as *Veillonella*, *Streptococcus*, *Prevotella*, and *Haemophilus*. The SPT profile is associated with an advanced stage and worse prognosis [6].

### Lung intratumor microbiota

The abundance of bacteria within tumors is low, with bacteria present in only 0.1%–10% of tumor cells. These bacteria often exist within the cytoplasm, with a much higher bacterial load in cancer cells compared with various immune and stromal cells [7]. Bacteria within lung cancer may originate from nearby bronchioles, with a small fraction potentially coming from the bloodstream.

The intratumor bacterial composition varies among tumors with different clinical features. Additionally, lifestyle influences the lung cancer microenvironment, thereby shaping the characteristics of the microbial community [7, 8]. For example, in lesions of smokers, the bacterial load is lower compared with nonsmokers, potentially due to smoking creating an unfavorable environment for bacterial growth. Bacteria isolated from lung cancer in smokers show enrichment of metabolic pathways capable of degrading chemicals [7].

## CARCINOGENIC MECHANISMS

### Direct carcinogenic effects on epithelial cells

#### DNA damages and chromosomal instability

Dysbiosis of microbiota can directly cause DNA damage, mutation, and genomic instability in epithelial cells through genotoxins or specific metabolites, thereby initiating or promoting the carcinogenic process. For example, aberrant microbiota can modify reactive oxygen species (ROS) levels, directly causing DNA damage in respiratory epithelial cells. Another classic example is a cytolethal distending toxin (CDT), produced by several Gram‐negative bacteria, which has the CdtB subunit that can be translocated into the nuclear compartment, exhibiting DNase I‐like activity and causing DNA double‐strand or single‐strand breaks [9].

#### Activation of signaling pathways

Oral bacteria, such as *Streptococcus* and *Veillonella*, induce the upregulation of extracellular signal‐regulated kinase (ERK) and phosphoinositide 3‐kinase (PI3K) signaling pathways in airway epithelial cells, which have been widely demonstrated to be involved in the initiation, metastasis, and therapy resistance of lung cancer [6]. Also, the expression levels of genes such as *CTNNB1* (involved in the Wnt/β‐catenin pathway), *HIF1A* (involved in hypoxia), and *VEGFA* (involved in angiogenesis) are positively correlated with the bacterial burden within lung cancer [8].

#### Interaction of metabolic processes

The abnormal composition of the microbiota can also affect the local metabolite network, with specific metabolites potentially participating in tumor cell metabolic processes, thus promoting tumor development. *Lactobacillus iners*, an obligate l‐lactate‐producing lactic acid bacterium found in tumors, can enrich tumor cells with metabolic pathways closely related to lactate signaling. Notably, lung cancers exhibit a marked preference for utilizing lactate over glucose to fuel the citric acid cycle. As expected, *Lactobacillus iners* in non‐small cell lung cancer (NSCLC) is closely associated with reduced recurrence‐free survival (RFS) [10]. However, experimental evidence in this field remains relatively scarce, necessitating further research in future studies.

### Excessive or unsolved immune activation

#### Microbial dysbiosis initiates pro‐carcinogenic inflammation via toll‐like receptors (TLRs)

TLRs serve as initiators of innate immune responses. Upon recognizing microbial components, these receptors induce the activation and translocation of nuclear factor‐κB (NF‐κB), activator protein 1 (AP‐1), and interferon regulatory factor 3 (IRF3) into the nucleus, resulting in the transcription of proinflammatory cytokines such as interleukin‐6 (IL‐6), tumor necrosis factor (TNF), and the inactive precursor of IL‐1β (pro‐IL‐1β) [11]. In fact, the expression of TLR‐4 and TLR‐9 has been shown to be upregulated in lung cancer.

#### Bridging role of proinflammatory cytokines

Segal et al. demonstrated that the presence of the SPT pneumotype microbiota is associated with enhanced expression of inflammatory cytokines such as IL‐1α, IL‐1β, IL‐6, fractalkine, and IL‐17 [12]. In the lungs, IL‐17A can induce the production of IL‐6 and granulocyte colony‐stimulating factor (G‐CSF), directly promoting tumor growth. It also facilitates tumor proliferation by recruiting tumor‐associated neutrophils and mediating resistance to immunotherapy. IL‐6 plays a crucial role in promoting lung cancer progression, metastasis, and drug resistance by upregulating the T‐cell immunoglobulin domain and mucin domain 4 (TIM‐4) via the NF‐κB pathway or by activating signal transducer and activator of transcription 3 (STAT3) and insulin‐like growth factor‐1 receptor (IGF‐1R) to induce epithelial‐to‐mesenchymal transition (EMT).

#### Neutrophils are recruited and promote tumor proliferation

The SPT pneumotype microbiota is associated with increased numbers of neutrophils in bronchoalveolar lavage fluid (BALF) [12]. Various cytokines produced following TLR activation can induce neutrophil recruitment. For example, IL‐17A can promote G‐CSF expression in lung tissue, resulting in the recruitment of neutrophils [13]. Recruited neutrophils, through the action of ROS, and reactive nitrogen species (RNS), create an environment favoring the transformation of lung cells to cancerous cells. *Mycobacterium tuberculosis* is a prime example of this carcinogenic process.

#### Key role of Th17 cells in pro‐carcinogenic inflammation

Th17 cells have been shown to play a critical role in the development of lung cancer [14]. IL‐6 and TGF‐β promote Th17 differentiation, and IL‐23 and IL‐21 subsequently maintain the Th17 lineage by enhancing the transcription of IL‐17. The SPT pneumotype is associated with a higher proportion of Th17 cells, as well as elevated levels of STAT3 and its downstream molecules. Through the production of IL‐17, Th17 cells can promote tumor cell proliferation and angiogenesis, as well as increase the recruitment of myeloid cells.

#### γδ T cells as critical pro‐inflammatory cells in carcinogenesis

Local microbes stimulate Myd88‐dependent IL‐1β and IL‐23 production from alveolar macrophages and neutrophils via TLRs, inducing the proliferation and activation of γδ T cells [13]. A significant portion of tumor‐enriched γδ T cells, which generate the proinflammatory cytokine IL‐17A, is defined as γδ T17 cells. Additionally, IL‐22, an epithelial growth factor promoting tumor cell proliferation in vitro and closely related to tumor metastasis, is significantly upregulated in lung‐tumor‐associated γδ T cells. Experimental evidence has indicated that eliminating the local microbiota or inhibiting γδ T cells or their downstream effector molecules effectively suppresses lung cancer development [13].

#### Immunosuppression

In response to excessive activation of innate immunity, epithelial cells may initiate transcriptional programs to dampen innate signaling, and Tregs and M2 macrophages are induced to limit inflammation. These changes may allow lung epithelial cells, which have already accumulated tumor characteristics, to evade immune surveillance and transform into tumor cells.

#### Alveolar macrophages (AMs) and M2 polarization

Some specific bacteria, such as *Staphylococcus aureus*, have been shown to have the ability to upregulate the expression of M2 macrophage markers such as CD206 and Dectin‐1 on AMs via TLR‐2 signaling. They also upregulate the expression of M2 macrophage‐associated genes, including *Arg1*, *Ym1*, *Fizz1*, *Il10*, and *Tgfb1*. Moreover, *Staphylococcus aureus* recruits peripheral blood monocytes to the alveoli, increasing the number of AMs and inducing their M2 polarization [15]. These M2‐polarized alveolar macrophages suppress immune responses through the secretion of anti‐inflammatory cytokines and the expression of inhibitory ligands.

#### Tregs

**Figure 2:**
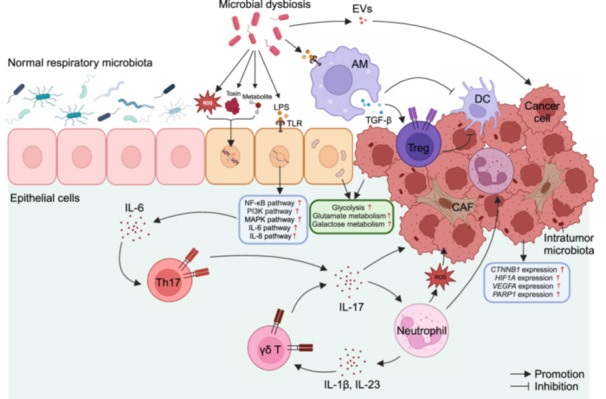
Mechanism diagram of microbial dysbiosis leading to lung epithelial carcinogenesis and promoting tumor development. An abnormal microbial composition can promote lung cancer through three mechanisms: direct carcinogenic effects, excessive or unsolved immune activation, and immunosuppression. AM, alveolar macrophage; CAF, cancer‐associated fibroblast; DC, dendritic cell; EV, extracellular vesicle; IL, interleukin; LPS, lipopolysaccharide; ROS, reactive oxygen species; TGF‐β, transforming growth factor‐β; TLR, toll‐like receptor; Treg, regulatory T cell.

Specific bacteria, such as *Bacteroides fragilis*, can directly promote the induction and accumulation of Tregs [16]. Short‐chain fatty acids (SCFAs), such as butyrate, which are intermediate products of anaerobic bacterial metabolism colonizing the lower respiratory tract, can expand the pool of Tregs through mechanisms including inducing *FOXP3* and *IL‐10* expression, modulating G‐protein‐coupled receptor signaling and the mammalian target of rapamycin (mTOR) signaling, and reshaping the T‐cell epigenome [17]. The mechanism diagram illustrates how microbes promote the initiation and progression of lung cancer (Figure 2).

## CLINICAL APPLICATIONS OF MICROBIOTA IN RESPIRATORY SYSTEM AND TUMOR

### Biomarker of prognosis

Utilizing the compositional characteristics of the lower respiratory tract or tumor microbiota in lung cancer patients can help predict prognosis and recurrence risk. The higher bacterial richness and diversity in the normal lung tissue of lung cancer patients, along with a higher abundance of *Lachnospiraceae*, *Faecalibacterium*, *Ruminococcus*, and *Roseburia*, are associated with a poorer prognosis, whereas a higher abundance of *Koribacteraceae* indicates a better prognosis [18]. In patients with stage IIIB‐IV lung cancer, the more similar the composition of the upper and lower respiratory tract microbiota, the higher the likelihood of progression [6].

### Enhancing treatment efficacy

The lung microbiome also holds the potential to enhance antitumor therapies in the future. Le et al. found that aerosolized *Lactobacillus rhamnosus GG* or *Bifidobacterium* significantly enhanced the efficacy of antitumor drugs [19]. In addition, there is evidence linking *Fusobacterium nucleatum* with poor immunotherapy efficacy in metastatic NSCLC [20]. Some scholars have also proposed the “gut–lung axis” hypothesis, suggesting that adjusting the gut microbiota can modulate immune cell differentiation, activation, and cytokine secretion, thereby affecting the respiratory system via the mucosal lymphatic system and bloodstream.

## CONCLUSION

Advances in sequencing technologies helped us identify previously unrecognized microbiota in the lower respiratory tract and within lung cancer. However, we need to note that the bacterial species identified in existing studies were not completely consistent. Therefore, rather than focusing on specific bacterial species, it is more important to focus on the functional enrichment and biological roles of these different microbial communities. Currently, it is unclear how these different microbes jointly or individually influence carcinogenic signaling pathways, and further exploration is needed to understand their interactions with other types of cells in the microenvironment. Dysbiosis of the lower respiratory tract microbiota drives lung cancer progression and is therefore a potential therapeutic target. Nevertheless, due to the low burden of pulmonary microbiota, the clinical value requires further investigation. Additionally, the safety of aerosolized bacteria needs to be carefully considered.

## AUTHOR CONTRIBUTIONS

**Zhihua Liu, Naixin Liang, and Shanqing Li:** Conceptualization; methodology. **Bowen Li and Daoyun Wang:** Investigation; writing—original draft; visualization. **Chengye Zhang and Yadong Wang:** Writing—review & Editing. **Zhicheng Huang, Libing Yang, and Huaxia Yang:** Data curation. All authors have read the final manuscript and approved it for publication.

## CONFLICT OF INTEREST STATEMENT

The authors declare no conflict of interest.

## ETHICS STATEMENT

No animals or humans were involved in this study.

## Data Availability

No new data and scripts were used for this commentary. Supplementary information (graphical abstract, slides, videos, Chinese translated version, and update materials) is available online DOI or http://www.imeta.science/.
